# Sensory impairments and subjective well-being status in middle-aged and older Chinese population: Cross-sectional and longitudinal analyses of a nationally representative survey

**DOI:** 10.3389/fpubh.2023.1099754

**Published:** 2023-03-16

**Authors:** Yifan Zhou, Yan Lu, Ye Liu, Yanan Hou, Xinyi Qian, Kaiweisa Abuduxukuer, Minhong Xiang, Qing Peng, Jianfeng Luo

**Affiliations:** ^1^Department of Ophthalmology, Putuo People's Hospital, Tongji University, Shanghai, China; ^2^Office of Party and Government Affairs, Healthcare Services Center, Yichuan Street Community, Putuo, Shanghai, China; ^3^Department of Clinical Medicine, School of Medicine, Soochow University, Suzhou, China; ^4^Department of Biostatistics, School of Public Health, Fudan University, Shanghai, China; ^5^NHC Key Laboratory of Health Technology Assessment, Fudan University, Shanghai, China; ^6^Key Laboratory of Public Health Safety of Ministry of Education, Fudan University, Shanghai, China; ^7^Department of Endocrine and Metabolic Diseases, Shanghai Institute of Endocrine and Metabolic Diseases, Ruijin Hospital, Shanghai Jiao Tong University School of Medicine, Shanghai, China; ^8^Department of Endocrine and Metabolic Diseases, Shanghai General Hospital (Shanghai First People's Hospital), Shanghai Jiao Tong University School of Medicine, Shanghai, China; ^9^Department of Geriatric Psychiatry, Shanghai Mental Health Center, Shanghai, China; ^10^Department of Ophthalmology, Putuo Hospital, Shanghai University of Traditional Chinese Medicine, Shanghai, China; ^11^Department of Ophthalmology, Shanghai Tenth People's Hospital, Tongji University, School of Medicine, Shanghai, China

**Keywords:** China health and retirement longitudinal study, life expectancy, life satisfaction, self-rated health, sensory impairment

## Abstract

**Purpose:**

To investigate the impacts of sensory impairments (SIs) including single vision impairment (SVI), single hearing impairment (SHI) and dual sensory impairment (DSI) on subjective wellbeing measurements including life expectancy (LE), life satisfaction (LS) and self-rated health (SRH) in middle-aged and older Chinese population.

**Methods:**

We obtained data from the China Health and Retirement Longitudinal Survey (CHARLS). In total, 9,293 Chinese middle-aged and older adults aging over 45 were included at baseline 2011 in this study, and 3,932 participants who accomplished all 4 interviews from 2011 to 2018 were adapted for longitudinal analyses. Sensory status and subjective wellbeing measurements were collected. Other covariates included socio-demographic characteristics, medical condition and lifestyle-related factors. The impacts of baseline sensory status on LE, LS and SRH were assessed using univariate and multivariate logistic regression analyses. A linear regression analysis with generalized estimating equations (GEE) was used to assess the association between time-varying sensory statuses with LE, LS and SRH over 8 years after being adjusted with multi-confounding factors.

**Results:**

Participants with SIs had significantly lower level of LE, LS, and SRH, compared to those who were free of SI. All kinds of SIs were significantly associated with LE, LS, and SRH according to cross-sectional data. The correlations between SIs and LE or SRH over 8 years were also noticed. However, only SHI and DSI were found to be significantly associated with LS according to longitudinal data (all *p* values < 0.05).

**Conclusion:**

Sensory impairments had explicitly detrimental effects on subjective wellbeing status over time among middle-aged and older Chinese population.

## 1. Introduction

The wellbeing status of aging population is one major challenge for both developed and developing countries all over the world (1). Substantial increase in the number of middle-aged and older people in our society generates an urgent requirement of attaining a successful aging life, which fosters daunting challenges for biological, social and medical science. Over the past decades, although the clear definition and scope of a successful aging have not reached a consensus, many researchers have emphasized the subjective nature of this concept and suggested that to define a successful aging, the self-perception of elderly individuals should be well considered (2).

Subjective life expectancy (LE), also known as perceived life expectancy or perception of future time (PFT), represents an individual's expectation or subjective probability of survival to a certain age, which has been considered as a pivotal predictor of one' s economic, health, and mental status and a direct benchmark survival indicator (3). On the other hand, the adage: “happy person lives longer” appears to be common knowledge. Life satisfaction (LS), defined as the perception of a person's overall quality of life, has also been regarded as an essential representative that reflects individual's overall subjective life evaluation (4). Also increasingly, self-rated health (SRH) has been used as a succinct way to approximate diverse objective components of health status, such as physical, psychological and behavioral health factors (5), which indicates the continuum of a person's perceived health by eliciting perspective of the individual on previous, current, and future health (6). These subjective wellbeing measurements have gained certain attention from the academic for evaluation of a successful aging, which have been widely adapted in studies on health and wellbeing status in recent years.

The judgements of subjective perceptions are usually based on personal criteria, which could be affected by situational factors under many circumstances (7). Therefore, identification of the affecting factors, especially modifiable risk factors of subjective wellbeing status may not only help revealing the inherent mechanisms of subjective elements of health and wellbeing status, but also provide supports of specific interventions to promote self-perception of a better living status. To date, multiple factors have been noticed to have certain impacts on subjective wellbeing status. For instance, individuals with higher age (8), pain and limitation of physical function (9) would be more likely to report poorer health status. Lower educational level, financial strain and lower depressive symptoms were reported to have strong associations with higher life satisfaction (10). And Personal socioeconomic status could greatly affect one's perception of future time (11). However, to the best of our knowledge, the effects of a common chronic physical dysfunction among middle-aged and older population, sensory impairment, have not been well explored (8).

With the increasing trend in life expectancy, sensory impairments (SIs), including hearing impairment (HI), vision impairment (VI), and dual sensory impairment (DSI) which refers to the simultaneous presence of VI and HI, are common age-related conditions. SIs have wide ranging implications for health and general wellbeing in elderly population (12), whose impacts on various adverse events in aging life including psychological distress (13), jeopardized quality of life (14), and even all-cause mortality (15–17) have been widely investigated. However, there is a paucity of literature on the associations between SI with subjective wellbeing measurements including LE, LS, and SRH in aing life, especially from developing countries (8, 18, 19).

China is the most populous developing country, which also faces severe aging society problems. More importantly, elderly Chinese are likely to neglect SIs and consequent problems owing to the traditional attitudes regarding SIs as normal parts of aging life, which might further contribute to the higher prevalence of SIs in China than in some western countries (20). SIs have been noticed to have certain influences on adverse health consequences in middle-aged and older Chinese population (21–23). Allowing for the specific cultural background, social institution, and health system in mainland China, the aim of the present study is to address the research gap of the associations between SIs with LE/LS/SRH among middle-aged and older Chinese population using cross-sectional and longitudinal data over 8 years of observation.

## 2. Methods

### 2.1. Participants and public involvement

Data was obtained from the China Health and Retirement Longitudinal Study (CHARLS). CHARLS is a longitudinal survey that aims to be representative of the residents in mainland China aged 45 and older. It attempts to set up a high quality public micro-database, which can provide a wide range of information from socioeconomic status to health conditions, to serve the needs of scientific research on the elderly. To ensure the adoption of best practices and international comparability or results, CHARLS is harmonized with leading international research studies in the Health and Retirement Study (HRS) model. The national baseline survey was conducted in 2011–12, with wave 2 in 2013, wave 3 in 2015, and wave 4 in 2018. In order to ensure sample representativeness, the CHARLS baseline survey covered 150 countries/districts, 450 villages/urban communities, across 28 provinces over the country, involving 17,708 individuals in 10,257 households, reflecting the mid-aged and older Chinese population collectively. With response rates over 80%, CHARLS provides the most up-to-date longitudinal data for investigation of the health status and wellbeing of middle-aged and elderly population in China. CHARLS enrolled 17,708 participants at baseline (Wave 1), 18,254 participants at Wave 2 (2013), 20,273 participants at Wave 3 (2015), and 19,816 participants at Wave 4 (2018). For respondents surveyed in the baseline wave, more than 90% of them were re-contacted in each of the follow-up waves, and the response rate of the tracked sample (panel sample) remains at higher than 86% in any of the follow-up waves. Therefore, the success follow-up rates of CHARLS are high compared to many HRS-type surveys.

## 3. Measures

### 3.1. Main outcome

#### 3.1.1. Life expectancy

In CHARLS, life expectancy was assessed according to the respondent's response to a series of stratified questions: “On what step (“Almost impossible”, “Not very likely”, “Maybe”, “Very likely”, “Almost certain”) do you think is your chance that you will live to be [75 (if respondent's current age is 64 or less)/80 (if age is between 65 and 69)/85 (if age is between 70 and 74)/90 (if age is between 75 and 79)/95 (if age is between 80 and 84)/100 (if age is between 85 and 89)/105 (if age is between 90 and 94)/110 (if age is between 95 and 99)/115 (if age is 100 or more)]?”. The 5-point Likert scale responses range from 1 (almost impossible) to 5 (almost certain). Higher SLE scores indicate longer lifespan anticipation. In this study, we defined SLE scores of 1–3 as lower life expectancy and considered SLE scores of 4–5 as higher life expectancy.”

#### 3.1.2. Life satisfaction

Life satisfaction was measured according to the respondent's response to the question “Please think about your life as a whole. Are you completely satisfied, very satisfied, somewhat satisfied, not very satisfied, or not at all satisfied with it?” Satisfaction is defined by answers including “completely satisfied”, “very satisfied” and “somewhat satisfied”, while dissatisfaction is defined by answers including “not very satisfied” and “not at all satisfied”.

#### 3.1.3. Self-rated health

Self-rated health status was acquired by the question: “Would you say your health is very good, good, fair, poor or very poor”. In our study, we defined SRH by dichotomizing answers into “very good to fair” vs. “poor to very poor” for subsequent analysis.

#### 3.1.4. Exposures

The main exposure in this present study is sensory status including no sensory impairment (NSI), single vision impairment (SVI), single hearing impairment (SHI) and dual sensory impairments (DSI). In CHARLS, VI consists of distal and near ones. Distal VI and near VI were evaluated by asking participants whether their eyesight was excellent, very good, good, fair, or poor when seeing things at a distance or up close, respectively. Reporting of fair or poor eyesight was classified as VI. Similarly, for HI assessment, the question was: “Is your hearing excellent, very good, good, fair or poor.” A response of fair or poor hearing was identified as HI. Such assessment of SI has been widely used in previous CHARLS-related studies (22). DSI refers to participants with both VI and HI, and single SI refers to sole VI or HI without the other one.

SI in aging population could be amended by medical supports such as cataract surgery and hearing aids, or vice versa, get worse due to aging or pathologic progressions. Thus, along with baseline sensory status, we also investigated the impacts of time-varying SI statuses during 8 years of follow-up on the outcomes to further explore the longitudinal effects on subjective wellbeing status.

### 3.2. Other variates

#### 3.2.1. Socio-demographic characteristics

Gender was a binary variable: male and female. Age was treated as a categorical variable including 3 groups (45–59, 60–74, and ≥75 years). Marital status indicated whether the respondent lived alone or got accompanied. Participants who were separated, divorced, widowed or never married were coded as “living alone', while those who were married or partnered were coded as “living with partner”. Living area referred to urban or rural places where participants lived. Educational attainment represents one's social economic status, which could probably affect people's access to health supports and other socio-economic resources. Educational status was categorized into 5 groups: illiterate, less than elementary school, elementary school, middle school, and high school or above.

#### 3.2.2. Medical condition

Data on the medical condition were collected with the following question: “Have you ever been diagnosed by a doctor as having the following diseases: hypertension, dyslipidemia, diabetes, cancer, chronic lung diseases, liver diseases, heart disease, stroke, kidney diseases, memory-related diseases, digestive diseases, arthritis, and asthma?”. Suffering from more than 2 diseases were defined as multi-morbidities. Insurance covering referred to coverage of one kind of health insurance or more.

#### 3.2.3. Lifestyle-related factors

The lifestyle variables included smoking and drinking status. Smoking is categorized as current/former smoker or never smoked. Drinking is a 3-category variable which indicates the frequency of drinking: none, less than once a month or more than once a month.

### 3.4. Statistical analysis

Statistical analyses were performed using SAS, version 9.4 (SAS Institute, Cary, NC, US). In this study, the primary exposures of interest were SIs, while the other independent variables served as control variables. Baseline characteristics were compared among participants according to SI statuses (4 groups) using the Chi-square test analysis. Logistic regression analyses were conducted to assess the associations between SIs, multiple covariates and subjective wellbeing measurements at baseline for cross-sectional analyses. Associations between time-variant SIs and all three subjective wellbeing measurements changes over time (across 4 interviews over 8 years) were assessed using logistic regression analysis with generalized estimating equations (GEE), controlling for the intraindividual correlation between repeated measurements using an exchangeable correlation structure as previously described (24). To examine whether LE, LS, and SRH were dependent on the status of sensory impairments, models that adjusted for potential confounders as mentioned above were employed, and their parameter estimates were shown with 95% confidence intervals.

## 4. Results

**Figure 1:**
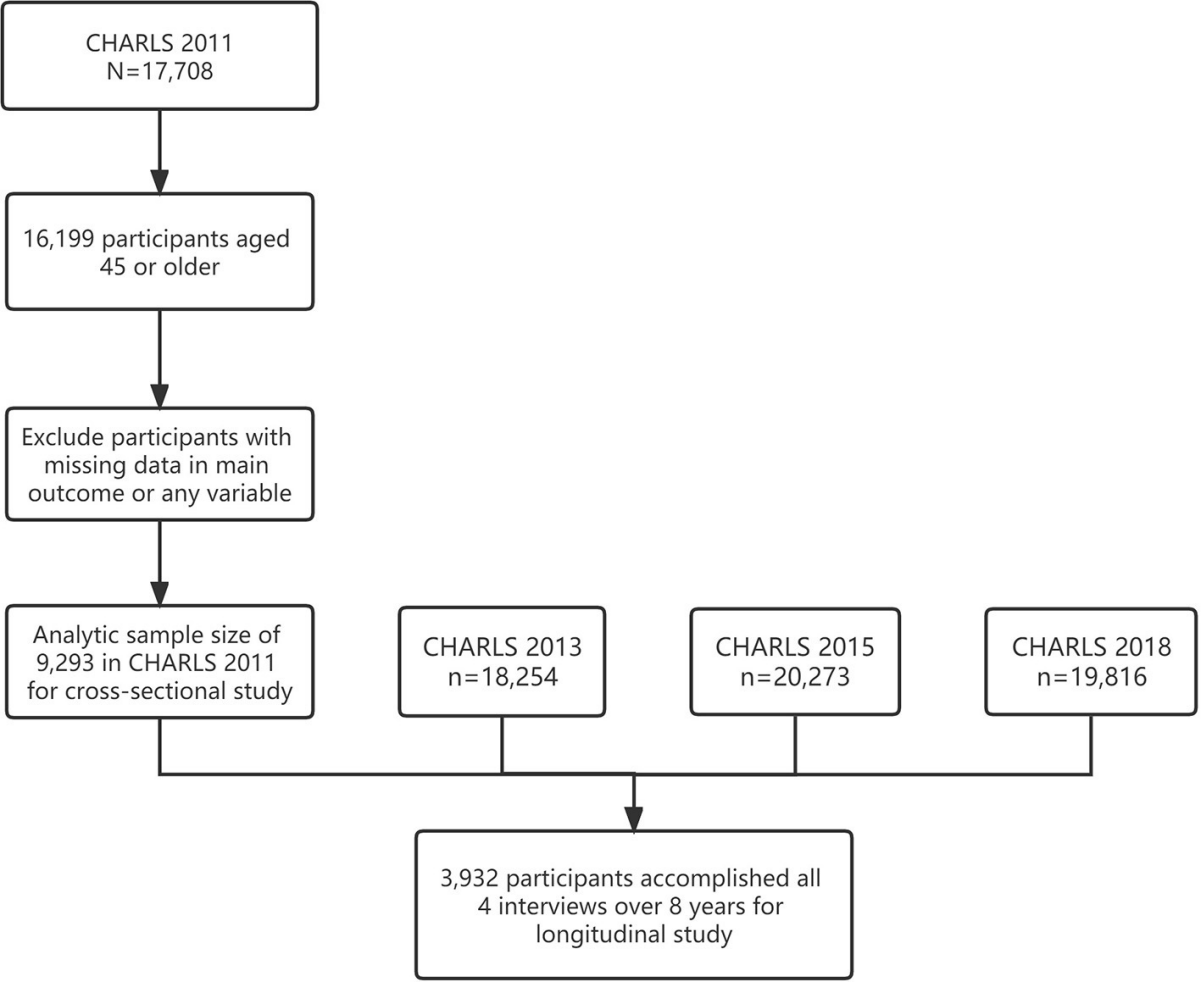
Graphic abstract of sample screening of the present study.

**Figure 2:**
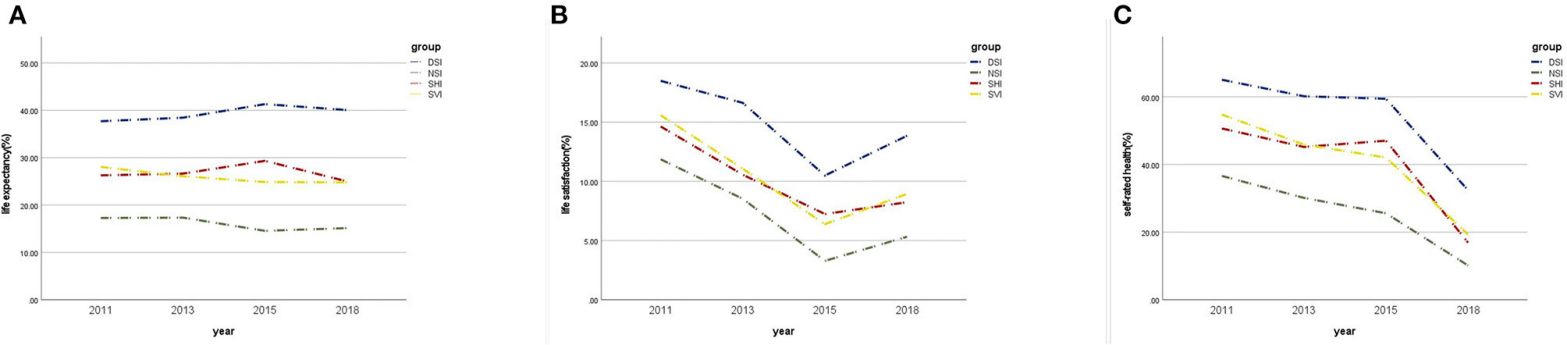
Changes of subjective wellbeing measurements over time, 2011–2018. **(A)** Changes in life expectancy by sensory impairment status; **(B)** Changes in life by satisfaction by sensory impairment status; **(C)** Changes in self-rated by sensory impairment status.

In total, 9,293 participants over 45 years old from baseline CHARLS 2011 were deemed eligible for the current study, among which 3,932 accomplished all 4 interviews from 2011 to 2018 and were adapted in the longitudinal analyses (Figure 1). The baseline socio-demographic characteristics, medical conditions, lifestyle-related factors and subjective wellbeing measurements of the study sample were grouped by sensory statuses and shown in Table 1. Participants who were free from any kind of sensory impairment appeared to have explicitly higher LE, LS, and SRH. Changes of LE, LS, and SRH over 8 years were performed in Figure 2.

**Table 1:** Characteristics of 9,293 participants of the present study sample from CHARLS 2011.

**Variables**	**Total**	**NSI**	**SVI**	**SHI**	**DSI**	***P*-value**
**Gender**						< 0.0001
Male	4,630 (49.82%)	1,600 (52.44)	577 (46.12)	867 (53.09)	1,586 (47.23)	
Female	4,663 (50.18)	1,451 (47.56)	674 (53.88)	766 (46.91)	1,772 (52.77)	
**Age**						< 0.0001
45–59	5,202 (55.98)	2,010 (65.88)	699 (55.88)	889 (54.44)	1,604 (47.77)	
60–74	3,415 (36.75)	908 (29.76)	469 (37.89)	613 (37.54)	1,425 (42.44)	
75–	676 (7.27)	133 (4.36)	83 (6.63)	131 (8.02)	329 (9.80)	
**Marital status**						0.0051
Live with partner	7,807 (84.01)	2,614 (85.68)	1,053 (84.17)	1,373 (84.08)	2,767 (82.40)	
Live alone	1,486 (15.99)	437 (14.32)	198 (15.83)	260 (15.92)	591 (17.60)	
**Education**						< 0.0001
Illiterate	2,244 (24.15)	606 (19.86)	329 (26.30)	363 (22.23)	946 (28.17)	
Less than elementary school	1,621 (17.44)	442 (14.49)	224 (17.91)	292 (17.88)	663 (19.74)	
Elementary school	2,053 (22.09)	617 (20.22)	276 (22.06)	383 (23.45)	777 (23.14)	
Middle school or vocational school	2,062 (22.19)	773 (25.34)	260 (20.78)	371 (22.72)	658 (19.59)	
High school and above	1,313 (14.13)	613 (20.09)	162 (12.95)	224 (13.72)	314 (9.35)	
**Living area**						< 0.0001
Urban area	3,865 (41.59)	1,447 (47.43)	530 (42.37)	640 (39.19)	1,248 (37.16)	
Rural area	5,428 (58.41)	1,604 (52.57)	721 (57.63)	993 (60.81)	2,110 (62.84)	
**Smoke**						0.0369
Yes	3,800 (40.89)	1,243 (40.74)	486 (38.85)	716 (43.85)	1,355 (40.35)	
No	5,493 (59.11)	1,808 (59.26)	765 (61.15)	917 (59.65)	2,003 (59.65)	
**Drinking status**						0.0009
Drink more than once a month	2,422 (26.06)	835 (27.37)	314 (25.10)	456 (27.92)	817 (24.33)	
Drink but less than once a month	727 (7.82)	269 (8.82)	80 (6.39)	128 (7.84)	250 (7.44)	
No drink	6,144 (66.11)	1,947 (63.82)	857 (68.51)	1,049 (64.24)	2,291 (68.23)	
**Multi-morbidities**						< 0.0001
Yes	1,803 (19.40)	374 (12.26)	235 (18.78)	319 (19.53)	875 (26.06)	
No	7,490 (80.60)	2,677 (87.74)	1,016 (81.22)	1,314 (80.47)	2,483 (73.94)	
**Insurance covering**						0.4308
Yes	8,714 (93.77)	2,873 (94.17)	1,162 (92.89)	1,535 (94.00)	3,144 (93.63)	
No	579 (6.23)	178 (5.83)	89 (7.11)	98 (6.00)	214 (6.37)	
**Life expectancy**						< 0.0001
Higher	6,720 (72.31)	2,524 (82.73)	900 (71.94)	1,204 (73.73)	2,092 (62.30)	
Lower	2,573 (27.69)	527 (17.27)	351 (28.06)	429 (26.27)	1,266 (37.70)	
**Life satisfaction**						< 0.0001
Satisfaction	7,876 (84.75)	2,689 (88.14)	1,056 (84.41)	1,394 (85.36)	2,737 (81.51)	
Dissatisfaction	1,417 (15.25)	362 (11.86)	195 (15.59)	239 (14.64)	621 (18.49)	
**Self-rated health**						< 0.0001
Healthy	4,480 (48.21)	1,934 (63.39)	566 (45.24)	806 (49.36)	1,174 (34.96)	
Unhealthy	4,813 (51.79)	1,117 (36.61)	685 (54.76)	827 (50.64)	2,184 (65.04)	

Before multivariate regression analysis, we first runed an univariate logistic regression which indicated certain covariables, including gender, age, marital status, educational level, living area, alcohol consumption and multi-morbidities, that could probably confound the relationship between SIs and subjective wellbeing status (Supplementary Table 1). To clarify the cross-sectional association between SIs and LE/LS/SRH, we analyzed their relevance by controlling for the covariates (Table 2). At baseline, all kinds of SIs showed profound and detrimental impacts on all three subjective wellbeing measurements after being adjusted for various confounders in all 4 Models (all *p-*values < 0.05). Compared to single SI, DSI had higher odds ratios, which suggests a potentially greater impact on subjective well-being status. The results shows that DSI is associated with lower LE (OR: 1.969, 95% CI 1.779–2.179, *p* < 0.001), lower LS (OR: 1.422, 95% CI 1.224–1.652, *p* < 0.001) and poorer SRH (OR: 2.018, 95% CI 1.833–2.223, *p* < 0.001).

**Table 2:** Cross-sectional logistic regression analyses of sensory impairments and subjective wellbeing measurements.

**Sensory status**	**Model 1**		**Model 2**		**Model 3**		**Model 4**	
**Ref: NSI**	**OR**	**95% CI**	**OR**	**95% CI**	**OR**	**95% CI**	**OR**	**95% CI**
**Life expectancy**								
SHI	1.707^***^	(1.477, 1.972)	1.631^***^	(1.410, 1.887)	1.534^***^	(1.322, 1.780)	1.458^***^	(1.255, 1.695)
SVI	1.868^***^	(1.600, 2.181)	1.781^***^	(1.953, 2.733)	1.676^***^	(1.429, 1.965)	1.612^***^	(1.372, 1.893)
DSI	2.898^***^	(2.578, 3.258)	2.682^***^	(2.382, 3.019)	2.447^***^	(2.168, 2.762)	2.240^***^	(1.982, 2.533)
**Life satisfaction**								
SHI	1.273^**^	(1.112, 1.583)	1.327^**^	(1.112, 1.583)	1.275^**^	(1.067, 1.523)	1.241^*^	(1.038, 1.483)
SVI	1.371^**^	(1.136, 1.655)	1.413^***^	(1.169, 1.706)	1.354^**^	(1.120, 1.638)	1.319^**^	(1.089, 1.596)
DSI	1.685^***^	(1.465, 1.938)	1.791^***^	(1.553, 2.064)	1.675^***^	(1.451, 1.934)	1.599^***^	(1.382, 1.849)
**Self-rated health**								
SHI	1.776^***^	(1.573, 2.007)	1.741^***^	(1.541, 1.958)	1.701^***^	(1.504, 1.925)	1.611^***^	(1.420, 1.828)
SVI	2.095^***^	(1.834, 2.395)	2.028^***^	(1.773, 2.319)	1.983^***^	(1.732, 2.270)	1.916^***^	(1.669, 2.199)
DSI	3.221^***^	(2.908, 3.568)	3.078^***^	(2.775, 3.414)	2.972^***^	(2.676, 3.300)	2.722^***^	(2.445, 3.029)

The results of the logistic regression models were expressed as odds ratios (OR) and 95% confidence intervals (CI). The analytic sample size was 9,293.

Model 1: adjusted for demographic factors including age and gender; Model 2: adjusted for factors in Model 1, as well as social-economic including marital status, educational level and living area; Model 3: adjusted for factors in Model 2, as well as life-style factors including smoking status and alcohol consumption; Model 4: adjusted for factors in Model 3, as well as medical condition including multi-morbidities and insurance covering.

^*^p < 0.05; ^**^p < 0.01; ^***^p < 0.001.

NSI, No sensory impairment; SVI, Single vision impairment; SHI, Single hearing impairment; DSI, Dual sensory impairment.

In longitudinal analyses, associations between time-variant SIs and subjective wellbeing status over time were assessed using linear regression analysis with generalized estimating equations, which could provide longitudinal observation of intraindividual correlations between repeated measurements. Consistent with cross-sectional analyses, we found that all kinds of SIs were significantly associated with LE and SRH, even after receiving adjustments of various confounders (Table 3, all *p-*values < 0.001). As for SI-LS correlations, only SHI and DSI remained to be significantly associated with LS. Participants with SHI (odds ratio [OR] 1.461, 95% CI 1.185–1.801; *p* < 0.001) or DSI (odds ratio [OR] 1.422, 95% CI 1.224–1.652; *p* < 0.001) were more likely to have lower LS compared with participants without SIs after adjustments. But we failed to find any profound correlation between SVI and LS in any of the 4 models.

**Table 3:** Longitudinal logistic regression analyses of time-varying sensory impairment statuses and subjective wellbeing measurements, 2011–2018.

**Sensory status**	**Model 1**		**Model 2**		**Model 3**		**Model 4**	
**Ref: NSI**	**OR**	**95% CI**	**OR**	**95% CI**	**OR**	**95% CI**	**OR**	**95% CI**
**Life expectancy**								
SHI	1.402^***^	(1.235, 1.592)	1.387^***^	(1.217, 1.580)	1.201^***^	(1.201, 1.577)	1.365***	(1.188, 1.567)
SVI	1.363^***^	(1.237, 1.5020	1.358^***^	(1.227, 1.502)	1.222^***^	(1.222, 1.512)	1.360^***^	(1.220, 1.516)
DSI	2.022^***^	(1.844, 2.217)	1.998^***^	(1.817, 2.198)	1.799^***^	(1.799, 2.195)	1.969^***^	(1.779, 2.179)
**Life satisfaction**								
SHI	1.462^***^	(1.196, 1.787)	1.486^***^	(1.212, 1.823)	1.472^***^	(1.196, 1.811)	1.461^***^	(1.185, 1.801)
SVI	1.099	(0.930, 1.297)	1.087	(0.920, 1.284)	1.078	(0.910, 1.277)	1.074	(0.905, 1.274)
DSI	1.476^***^	(1.277, 1.706)	1.472^***^	(1.272, 1.704)	1.441^***^	(1.242, 1.672)	1.422^***^	(1.224, 1.652)
**Self-rated health**								
SHI	1.757^***^	(1.542, 2.002)	1.747^***^	(1.531, 1.992)	1.724^***^	(1.508, 1.970)	1.715^***^	(1.496, 1.965)
SVI	1.265^***^	(1.265, 1.555)	1.391^***^	91.253, 1.544)	1.387^***^	(1.247, 1.542)	1.401^***^	(1.258, 1.560)
DSL	1.927^***^	(1.927, 2.321)	2.077^***^	(1.891, 2.281)	2.035^***^	(1.849, 2.239)	2.018^***^	(1.833, 2.223)

The results of the logistic regression models were expressed as odds ratios (OR) and 95% confidence intervals (CI). The analytic sample size was 3,932.

Model 1: adjusted for demographic factors including age and gender; Model 2: adjusted for factors in Model 1, as well as social-economic including marital status, educational level and living area; Model 3: adjusted for factors in Model 2, as well as life-style factors including smoking status and alcohol consumption; Model 4: adjusted for factors in Model 3, as well as medical condition including multi-morbidities and insurance covering.

^*^p < 0.05; ^**^p < 0.01; ^***^p < 0.001.

NSI, No sensory impairment; SVI, Single vision impairment; SHI, Single hearing impairment; DSI, Dual sensory impairment.

## 5. Discussion

To date, few studies have examined the associations between sensory impairments and subjective wellbeing status among middle-aged and older population. To our knowledge, the present study provides explicit evidences on such associations from a national level survey among Chinese population for the first time, which extends current knowledge regarding this issue.

### 5.1. Life expectancy

LE has been conceived as a mental model of one's remaining lifetime, and the length of LE is related to physical conditions and mortality in later life (25). On the other hand, people form LE based on the rational expectations of remaining years, which could be possibly influenced by life experiences and perceptions of being (26). Thus, it is essential to investigate affecting factors of LE for prevention of inappropriate estimation of remaining life among aging population. To date, very few studies have mentioned the potential effects of some unmodifiable factors such as demographic characteristics including age and gender (27, 28), while the present study might be one of the very first studies that focused on the impacts of one common, modifiable and age-related physical disorder: sensory impairments, on the subjective life expectancy among middle-aged and older population.

Although SIs have been commonly considered to have strong associations with physical and mental health statuses in elderly people, which could possibly contribute to jeopardized life expectancy and increased mortality (29, 30), researches on the associations between SIs and LE have yielded mixed results over the past decades. Jagger et al. pointed out that both VI and HI reduced disability-free life expectancy (DFLE) among a sample of English population over 65 years old (31). However, total life expectancy (TLE) was not associated with HI, and VI-TLE associations was not observed in male population from that study (31). Another study based on the Australian Longitudinal Study of Aging (ALSA) and the Blue Mountains Eye Study (BMES) indicated that SIs greatly reduced TLE in Australian people over 65 years old (32). As for Chinese population, according to the present study, for the first time, we revealed that all kinds of SIs were significantly associated with LE in univariate logistic analysis. And after adjustment of multiple confounders in all 4 models, SVI, SHI and DSI were also significantly and independently associated with LE in both cross-sectional and longitudinal analyses. Thus, the present study showed light to the direct associations between SI and LE among Chinese population. We also propose future studies on underlying mechanisms of SI-LE associations and specific interventions.

### 5.2. Life satisfaction

According to our cross-sectional analyses, all SIs were found to be significantly associated with LS in univariate logistic regression. Such associations remained even after receiving adjustment of various confounders. These findings were consistent to two cross-sectional-designed studies among German people and French-speaking adults in eastern Canada (33, 34). We also noticed another relating study which reported cross-sectional SVI-LS association among very old Chinese population (over 95 years of age) (35). However, when we further tried to verify SIs-LS correlations in the longitudinal analyses, we failed to find any significant result of SVI-LS correlations in any of the 4 models. Our results provide important evidences that HI, especially along with concurrent VI, have significant impacts on LS, which would decline as a consequence of sensory deficits among middle-aged and older population.

Several hypotheses may explain why SI and life satisfaction are consistently associated. First of all, SI is associated with consequently decreased functional abilities (36). The mediating role of functional ability between SIs, especially visual deficit, and life satisfaction has been described in a previous study (35). Another reason that could underlie the link between SIs and life satisfaction might be negative health comparisons. That means individuals with SIs may compare their health status (especially sensory functions) with other individuals who are presumably free of SIs. Appearance of SIs may lead to conclusion of poorer health conditions compared to others. A previous study demonstrated that negative health comparisons could markedly reduce subjective wellbeing (37). Also, senior adults with SIs commonly need assistance from others to deal with daily activities (36, 38). It may seem impossible to repay the person whom they receive assistance from, which could have consequences for social relationships (39–41). People with SIs may perceive their relationships as unidirectional rather than bidirectional, and this feeling on dependency has been demonstrated to be associated with lower autonomy (41, 42).

The present study further indicated more profound longitudinal SHI-LS association than SVI-LS association, which was not consistent to the comparisons of OR values according to logistic regressions. One possible explanation for such situation might lay in the fact that there is less feasibility and probability in amelioration of SHL status than SVL status over time among aging Chinese. Assistive devices such as glasses and portable magnifiers are relatively efficient, economic and easy-to-carry interventions for quite a lot people who suffer reversible SVI (43). On the other hand, interventions like wearing hearing aids for improvement of hearing status would more likely confront financial constraints, unfamiliarity with hearing aids, and difficulties during manipulating, which might all contribute to the fact that application level of hearing aids is far less than expected among Chinese population (44). Besides amplifying desired sounds, hearing aids would amplify noises as well, thus making users feel too loud and noisy. Such muffled effect also jeopardizes peoples' belief in hearing aids (44). Some authors argued that, even when listening aids are used, the promotion of LS might be limited in people with HI (34, 45). Therefore, untreated HI and the subsequent sustaining hearing deficit might exert more explicitly longitudinal effects on LS among our population over time.

### 5.3. Self-rated health

Self-rated health is one of the various health concepts that have been developed from the definition of health according to the WHO statement in 1948 (46). Although the assessment of SRH is usually summarized into one general question, it has been broadly adapted as an indicator to monitor the health status of individuals (47), and it is often used as a screening tool in epidemiological surveys or for identifying persons at risk of diseases (47, 48).

In the present study, we provide explicit evidences of profound correlations between SIs and SRH, according to both cross-sectional and longitudinal analyses among middle-aged and older Chinese population. Similarly, the Cache County Investigators also reported that, among various health measurements, sensory impairment, including vision and hearing could be regarded as predictors of SRH (49). Longitudinal evidences were also raised from the Second Supplement on Aging (SOA-II) Study among American population (50). On the other hand, Harada et al. reported that self-perceived poor health only showed an association with VI but not with HI in rural Japanese people with older age (51). Astrid et al. also failed to identify SIs as risk factors among a Norwegian population of older people participating in a preventive home visit program, which is partly owing to selection bias and the vague definition of SIs in their study (9).

Factors that might increase the one's perceptions of good health appear to be broadly related to social participation. Studies have also shown that social factors, including social support, household composition, contact with family members and attending educational or training courses, are positively associated with better SRH (52, 53). Preserved hearing and visual functions facilitate social participation, which further improve social functioning and relationships. Old people who see their friends and relatives as often as they like are also more likely to rate their overall health positively (49, 50). On the other hand, SIs, including VI and HI, and more severe condition of DSI, have already been considered to have correlations with various adverse events in aging life, such as multiple chronic medical conditions (54), reduced physical activities (28), increased fall risk (22) and poorer mental health status (21). Each of these events, if sustained for extended periods, could adversely shape health outcomes, and eventually influence personal perception on health status.

Our findings clearly demonstrated the detrimental effects of vision and hearing impairments on SRH, a holistic subjective measure of individual health among middle-aged and older Chinese population. Due to the central role of SRH in age-related challenges and risks, we anticipate that new knowledge of SI-SRH association could facilitate the improvement of health status in aging life.

### 5.4. Strengths and limitations

CHARLS is a national study with a large sample size, indicating that the findings from the current study could be generalized to the entire country. to our knowledge, the current study is the first nation-wide study based on middle-aged and older Chinese population to verify the associations between SIs and subjective wellbeing status according to both cross-sectional study and longitudinal observation over 8 years. Results in our study could be used as a reference to promote living status among aging population in China. Lastly, multiple associated factors were included and adjusted in analyses, which could otherwise potentially confound the relationship between SIs and outcomes.

Meanwhile, we acknowledge some limitations. First, self-reports provide information about subjective perception, and therefore, are clinically highly relevant and suitable for studies on subjective wellbeing status like ours. Although this method has also been used in quite a few well-known population-based studies such as NHANES and NHIS, we still need to notice the potential bias owing to the nature of self-reported data. On the other hand, exclusion criteria of the present study might lead to selection bias. Second, although we tried best to adjust for as many covariates as possible, still other covariates that may affect subjective perceptions of wellbeing might not have been included in the present study. Future researches may consider more robust study designs such as randomized controlled trials for more reliable results.

## 6. Conclusion

In summary, the present study provides explicit evidences that sensory impairments including vision impairment, hearing impairment and dual sensory impairment are negatively associated with life expectancy, life satisfaction and self-rated health among middle-aged and older Chinese population.

## Data availability statement

Publicly available datasets were analyzed in this study. This data can be found here: The original dataset of CHARLS is accessible on http://charls.pku.edu.cn/.

## Ethics statement

The studies involving human participants were reviewed and approved by Institutional Review Board at Peking University. The patients/participants provided their written informed consent to participate in this study.

## Author contributions

QP and JL designed the research. KA, XQ, and YeL analyzed the data. YZ drafted the manuscript. YanL, MX, and YH revised the manuscript. YZ, YanL, and YeL contributed equally to this research and should be considered as equivalent authors. All authors read and approved the final manuscript.
