# Supplementary material for: Sensory impairments and subjective well-being status in middle-aged and older Chinese population: Cross-sectional and longitudinal analyses of a nationally representative survey
Source: Front Public Health. 2023 Mar 16;11:1099754. doi: 10.3389/fpubh.2023.1099754 (PMC10064882; doi:10.3389/fpubh.2023.1099754)
Supplement: Supplementary file 1 [file Table_1.docx]

Supplementary table 1. Univariate regression analyses of dependent variates and subjective well-being measurements in 2011

|  | Life expectancy | | Life satisfaction | | Self-rated health | |
| --- | --- | --- | --- | --- | --- | --- |
| Variables | OR (95% CI) | P value | OR (95% CI) | P value | OR (95% CI) | P value |
| Gender |  | |  | |  | |
| Male | Reference | | | | | |
| Female | 1.256(1.147,1.376) | ＜0.0001 | 1.139(1.017,1.275) | 0.0245 | 1.300(1.198,1.410) | ＜0.0001 |
| Age |  | |  | |  | |
| 45-59 | Reference | | | | | |
| 60-74 | 1.379(1.252,1.520) | ＜0.0001 | 0.772(0.683,0.872) | ＜0.0001 | 1.369(1.255,1.493) | ＜0.0001 |
| 75- | 2.630(2.232,3.100) | ＜0.0001 | 0.704(0.554,0.894) | 0.0041 | 1.493(1.269,1.756) | ＜0.0001 |
| Marital status |  | |  | |  | |
| Live with partner | Reference | | | | | |
| Living alone | 1.466(1.302,1.650) | ＜0.0001 | 1.392(1.206,1.607) | ＜0.0001 | 1.157(1.035,1.294) | 0.0102 |
| Education |  | |  | |  | |
| Illiterate | Reference | | | | | |
| Less than elementary school | 0.785(0.687,0.897) | ＜0.0001 | 0.995(0.842,1.174) | 0.9497 | 0.960(0.844,1.092) | 0.533 |
| Elementary School | 0.575(0.505,0.654) | 0.0004 | 0.733(0.621,0.864) | 0.0002 | 0.824(0.731,0.930) | 0.0017 |
| Middle school or vocational school | 0.396(0.346,0.454) | ＜0.0001 | 0.747(0.634,0.880) | 0.0005 | 0.642(0.569,0.724) | ＜0.0001 |
| High school and above | 0.251(0.210,0.300) | ＜0.0001 | 0.545(0.444,0.669) | ＜0.0001 | 0.548(0.477,0.629) | ＜0.0001 |
| Living area |  | |  | |  | |
| Urban area | Reference | | | | | |
| Rural area | 2.009(1.823,2.214) | ＜0.0001 | 1.471(1.306,1.657) | ＜0.0001 | 1.338(1.231,1.453) | ＜0.0001 |
| Smoke |  | |  | |  | |
| No | Reference | | | | | |
| Yes | 0.991(0.903,1.087) | 0.8458 | 0.985(0.878,1.105) | 0.7953 | 0.929(0.856,1.010) | 0.0828 |
| Drinking status |  | |  | |  | |
| No drink | Reference | | | | | |
| Drink but less than once a month | 0.653(0.543,0.785) | ＜0.0001 | 0.835(0.669,1.041) | 0.1094 | 0.694(0.595,0.810) | ＜0.0001 |
| Drink more than once a month | 0.720(0.646,0.802) | ＜0.0001 | 0.791(0.690,0.906) | 0.0007 | 0.622(0.566,0.684) | ＜0.0001 |
| Multi-morbidities |  | |  | |  | |
| No | Reference | | | | | |
| Yes | 2.481(2.229,2.762) | ＜0.0001 | 1.444(1.263,1.650) | ＜0.0001 | 3.627(3.228,4.076) | ＜0.0001 |
| Insurance covering |  | |  | |  | |
| No | Reference | | | | | |
| Yes | 1.071(0.885,1.295) | 0.4833 | 0.647(0.525,0.796) | ＜0.0001 | 1.158(0.979,1.370) | 0.0878 |
| Sensory status |  | |  | |  | |
| No sensory impairment | Reference | | | | | |
| Hearing impairment | 1.707(1.477,1.972) | ＜0.0001 | 1.273(1.068,1.518) | 0.0071 | 1.776(1.573,2.007) | ＜0.0001 |
| Vision impairment | 1.868(1.600,2.181) | ＜0.0001 | 1.371(1.136,1.655) | 0.001 | 2.095(1.834,2.395) | ＜0.0001 |
| Dual sensory impairment | 2.898(2.578,3.258) | ＜0.0001 | 1.685(1.465,1.938) | ＜0.0001 | 3.221(2.908,3.568) | ＜0.0001 |
